# Comprehensive Analysis of Differentially Expressed Profiles of mRNA 5-Methylcytosine Modification in Metabolic Dysfunction-Associated Steatotic Liver Disease

**DOI:** 10.3390/cimb47050305

**Published:** 2025-04-26

**Authors:** Yueying Yang, E Wang, Bing Zhou, Yan Lu, Xiaoying Ding, Yao Li

**Affiliations:** 1 Department of Endocrinology and Metabolism, Shanghai General Hospital, Shanghai Jiao Tong University School of Medicine, Shanghai 200080, China; yangyueying@sjtu.edu.cn; 2 Department of Laboratory Animal Science, Shanghai Jiao Tong University School of Medicine, 280 South Chongqing Road, Shanghai 200025, China; wange@shsmu.edu.cn; 3 Institute of Metabolism and Regenerative Medicine, Digestive Endoscopic Center, Shanghai Sixth People’s Hospital Affiliated to Shanghai Jiao Tong University School of Medicine, Shanghai 200233, China; zhoubingice@sjtu.edu.cn (B.Z.); luyan5011@shsmu.edu.cn (Y.L.); 4 Shanghai Key Laboratory of Diabetes Mellitus, Shanghai Diabetes Institute, Shanghai Clinical Centre for Diabetes, Shanghai Sixth People’s Hospital Affiliated to Shanghai Jiao Tong University School of Medicine, Shanghai 200233, China

**Keywords:** 5-methylcytosine, lipid metabolism, metabolic dysfunction-associated steatotic liver disease

## Abstract

RNA methylation plays a critical role in regulating all aspects of RNA function, which are implicated in the pathogenesis of various human diseases. Recent studies emphasize the role of 5-methylcytosine (m5C), an RNA modification, in key biological functions. Metabolic dysfunction-associated steatotic liver disease (MASLD) has emerged as the leading chronic liver condition globally. However, the relationship between m5C methylation and MASLD remains unclear. This study aimed to investigate m5C modification in a mouse model of MASLD. In this research, using RNA transcriptome sequencing (RNA-Seq) and methylated RNA bisulfite sequencing (RNA-BS-Seq) in leptin receptor-deficient mice, we found that genes associated with hypermethylation were primarily linked to lipid metabolism. We identified 156 overlapping and differentially expressed genes (DEGs) that changed at both the mRNA expression level and the m5C modification level. Among them, 72 genes showed elevated expression and m5C modification. Gene Ontology (GO) and Kyoto Encyclopedia of Genes and Genomes (KEGG) enrichment analyses indicated that these genes were significantly associated with lipid metabolism-related signaling pathways. Our results demonstrate that m5C methylation modifications may play an important role in the development of MASLD.

## 1. Introduction

Metabolic dysfunction-associated steatotic liver disease (MASLD) is the revised nomenclature of non-alcoholic fatty liver disease (NAFLD) [1]. MASLD has rapidly emerged as the most prevalent chronic liver disease worldwide, affecting approximately 38% of adults globally [2]. This condition includes two primary pathological states: metabolic dysfunction-associated steatotic liver (MASL) and metabolic dysfunction-associated steatohepatitis (MASH) [3]. As MASLD progresses to MASH, the risk of liver fibrosis, cirrhosis, and hepatocellular carcinoma (HCC) escalates, significantly increasing the associated morbidity and mortality. MASLD is now recognized as a multifaceted disorder influenced by multiple interacting risk factors, including genetic predispositions, metabolic dysfunctions, and environmental exposures. The progression of the disease involves complex mechanisms, such as the “second hit” theory, where initial lipid accumulation in liver cells triggers a cascade of cytotoxic events, leading to inflammation and cellular damage. Additionally, the “multiple hits” hypothesis suggests that genetics, metabolic syndrome, gut microbiota, and other factors collectively contribute to hepatic steatosis and inflammation. Despite advances in understanding these mechanisms, the precise pathogenesis of MASLD remains elusive [4,5]. Although research has improved diagnostic approaches, the mechanisms driving MASLD progression and effective treatments remain limited [6,7]. Consequently, there is an urgent need for more comprehensive and meticulous research to elucidate the underlying causes of the disease, develop accurate animal models, and identify targeted therapeutic interventions. These efforts are crucial for discovering viable molecular targets and treatment strategies that can be effectively integrated into practical medical applications [8].

Emerging research has established the pivotal role of RNA methylation in regulating a diverse range of biological processes [9]. Notably, advances in epitranscriptomics have revealed that disruptions in regulatory mechanisms, including RNA methylation, are intimately linked to cellular functions and the pathogenesis of numerous diseases [10,11]. As a post-transcriptional modification, RNA methylation influences gene expression by regulating RNA metabolism, including translation, splicing, and stability [12,13]. Among these modifications, m6A methylation modifications are widespread in eukaryotes and are also one of the most common and studied forms of RNA methylation modification. Building on this foundation, our research group has been consistently dedicated to investigating the role of RNA methylation in the pathogenesis of MASLD. We recently found that RPN11, a deubiquitinating enzyme, deubiquitinates and stabilizes METTL3, thereby enhancing the m6A modification of ACSS3. This modification could alleviate MASLD and MASH in mice [14]. Moreover, we identified IGF2BP2, an N6-methyladenosine modification reader, as a critical driver of MASH progression in mice and Ythdc2, an m6A reader, as an important factor in the regulation of hepatic lipogenesis and TG homeostasis [15].

Recently, m5C has attracted increasing research attention. However, the relationship between m5C methylation and MASLD remains largely unexplored. This epigenetic marker, found within RNA molecules, serves as a key regulator in processes such as RNA structural integrity, alternative splicing, and translational control. By interacting with specific recognition proteins, m5C mediates its biological effects and has been linked to various disease states, particularly in tumor development [16,17]. In eukaryotic systems, the addition of m5C to mRNA is primarily mediated by the NOP2/Sun domain (NSUN) family [18,19,20,21], while tRNA aspartic acid methyltransferase 1 (TRDMT1) predominantly targets tRNA [22,23]. This methylation process involves the modification of the fifth nitrogen atom of cytosine and is observed across diverse RNA species [13,24]. The dynamic process of m5C methylation formation is orchestrated by methyltransferases (“Writers”) and is counterbalanced by demethylases (“Erasers”) like TET families, along with binding proteins (“Readers”) such as YBX [25]. Much research has firmly demonstrated that m5C regulators play a crucial role in a diverse range of fundamental biological and pathological processes. These include not only cell growth and specialization but also the regulation of stem cell destiny, embryogenesis, the onset of cancer, its advancement, and the immune response within tumors [9,26]. Nevertheless, the potential role of RNA 5-methylcytosine in hepatic triglyceride metabolism regulation and its contribution to obesity-related metabolic dysfunction-associated steatotic liver disease remains an open question in the field. Recent studies highlight multi-omics approaches for investigating disease mechanisms [27,28,29]. A study explored the molecular signature of steatosis-to-MASH progression through large-scale system biology approaches. Integrating multiple types of omics datasets into a single network identified several new potential genes, proteins, metabolites, and non-coding RNAs associated with steatosis-to-MASH progression, providing a valuable resource for exploring the molecular mechanisms of MASH progression [30]. In this research, we used RNA transcriptome sequencing and methylated RNA bisulfite sequencing to explore the relationship between MASLD and m5C RNA methylation.

For this investigation, we employed *db*/*db* mice, which lack functional leptin receptors, as a representative model for MASLD. Our research demonstrates the critical role of m5C RNA methylation in influencing the progression of MASLD.

## 2. Materials and Methods

### 2.1. Animal Studies and Sample Collection

We used BKS-*db*/*db* (*db*/*db*) mice as a representative model for MASLD. The strain background of the *db*/*db* mice is C57BLKS/J (BKS). The *db*/*db* mice are homozygous mice. The leptin receptor gene (Lepr) in these mice has undergone a mutation, resulting in abnormal leptin signaling and the manifestation of symptoms similar to human type 2 diabetes, such as obesity, insulin resistance, and hyperglycemia. *db*/*wt* and *db*/*wt* mice mated to obtain *db*/*db* mice and lean mice (*wt*/*wt*). The littermate lean mice (*wt*/*wt*) were used as the normal control (NC) group because they have the same genetic background, breed, and strain as the *db*/*db* mice. Four male *db*/*db* mice and four male *wt*/*wt* mice aged 7 weeks were obtained from GemPharmatech company (Nanjing, China) and maintained in a controlled environment with a temperature of 21 ± 1 °C, relative humidity of 55% ± 10%, and a 12 h light/dark regimen. All the mice were fed a regular diet for one week for acclimation and then sacrificed at the eighth week. Liver samples were harvested and either flash-frozen in liquid nitrogen or preserved for subsequent pathological analysis. Tissue sections were embedded for hematoxylin and eosin (H&E) staining and oil red O staining. Plasma was collected and spun at 2500× *g* for 10 min, and blood samples were separated for additional analysis.

### 2.2. Liver Lipid Analysis and Biochemical Assessment

To assess plasma total cholesterol (TC), triglycerides (TG), alanine aminotransferase (ALT), and aspartate aminotransferase (AST), a ready-to-use kit from Kehua in Shanghai, China, was employed. The procedure was meticulously followed as per the guidelines. For the liver TG analysis, approximately 1 mg (liver)/50 µL (5% NP40 solution) was homogenized. The homogenate was then heated in a water bath at 70 °C for 10 min and centrifuged at 12,000× *g* for 5 min. The supernatant was collected, and TG content was quantified using the Triglyceride Quantification Kit from BioVision in Milpitas, CA, USA. There were four samples in each group. Each experiment was repeated three times.

### 2.3. RNA Isolation and Preparation

Total RNA was extracted from the livers of four *db*/*db* mice and the livers of four NC mice, respectively. According to the manufacturer’s protocol, the TRIzol reagent (Invitrogen Corporation, CA, USA) was used for the RNA extraction from livers. The liver RNA of each mouse was separately subjected to RNA transcriptome sequencing and methylated RNA bisulfite sequencing.

### 2.4. RNA-BS-Seq, RNA-Seq, and Data Analysis

Briefly, rRNA was eliminated from total RNA with the rRNA Removal Kit, or mRNA was enriched using the mRNA Enrichment Kit (Illumina, San Diego, CA, USA). The removed rRNA or enriched mRNA was transformed with bisulfite using the EZ RNA methylation Kit (Zymo Research, CA, USA). RNA strand-specific library construction was performed on the transformed samples. The libraries were qualified with a fragment analyzer and qubit quality control and then subjected to second-generation high-throughput sequencing. The quality of the libraries was assessed using the BioAnalyzer 2100 system from Agilent Technologies, Inc., based in Palo Alto, CA, USA. Sequencing was performed on an Illumina NovaSeq 6000 platform (Illumina, San Diego, CA, USA), generating 150 bp paired-end reads. Briefly, paired-end reads were harvested from the Illumina NovaSeq 6000 sequencer (Illumina, San Diego, CA, USA) and were quality-controlled with Q30. Low-quality reads and 3’ adapters were trimmed using the cutadapt software (version 1.9.3). Cleaned reads from the input libraries were then mapped to the reference genome (MM10) using Hisat2 (version 2.0.4). Subsequently, MACS software (version 1.4.2) was employed to compare these reads against the mouse genome, pinpointing RNA methylation sites (peaks) [31]. The detection of methylated regions on RNAs, referred to as peaks, was carried out using MACS software [32]. To pinpoint differentially methylated m5C sites (DMMSs), the diffReps tool (version 1.55.3) was employed [33]. Custom-built scripts were then utilized to analyze and isolate peaks identified by both programs that coincided with mRNA exons. Differentially methylated regions with a fold change ≥2 and *p* value ≤0.0001 and methylation sites that are specific only in a certain set of data were detected using the diffReps analysis tool. The RNA-Seq data and RNA-BS-Seq data are uploaded in the GEO. The accession numbers are GSE185228 and GSE291880, respectively.

### 2.5. RT-qPCR Validation

Total RNA was reverse-transcribed into cDNA using the Reverse Transcription System (Promega, Madison, WI, USA). Real-time qPCR (RT-qPCR) was carried out using the LightCycler 480 Instrument II (Roche Diagnostics, Basel, Switzerland) with SYBR Green Premix Ex Taq (Takara Bio, Shiga, Japan). Gene expression levels were relatively quantified using the comparative 2-ΔΔCt method and normalized to the endogenous reference gene 36B4. All primer sequences used for RT-qPCR analysis are provided in Supplementary Table S1.

### 2.6. Statistical Analysis

All data were analyzed using GraphPad Prism (version 9.0.0). The normality of all measurement data was first tested using the Shapiro–Wilk (S-W) test, and all the data conformed to a normal distribution. Subsequently, differences between the two groups were analyzed using Student’s *t*-test. Every statistic was shown as mean ± SEM. Significant differences were assumed when *p* < 0.05.

## 3. Results

### 3.1. db/db Mice as a Model of MASLD

**Figure 1:**
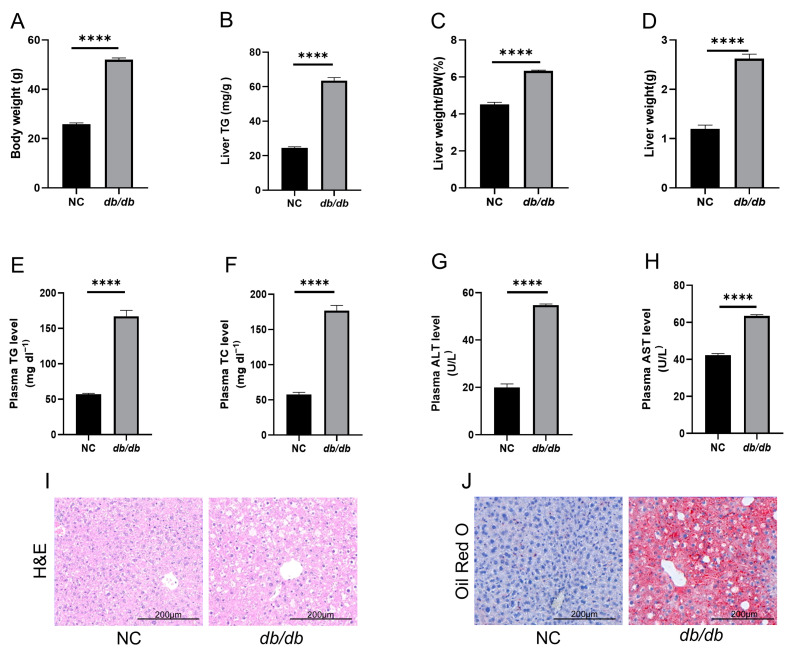
*db*/*db* mice as a model of MASLD: (**A**) Body weight. (**B**) Liver TG levels. (**C**) Liver weight/body weight. (**D**) Liver weight. (**E**) Plasma TG levels. (**F**) Plasma TC levels. (**G**) Plasma ALT levels. (**H**) Plasma AST levels. (**I**) H&E-stained liver sections. (**J**) Oil Red O-stained liver sections. *n* = 4 biologically independent mice per group. Data are expressed as mean ± SEM. **** *p* < 0.0001.

Our study demonstrated that *db*/*db* mice exhibited a significant increase in body weight compared to the NC group. Additionally, both liver weight and its proportion to the total body weight, along with liver TG levels, were markedly elevated in the *db*/*db* group. Plasma TG, TC, ALT, and AST levels also showed significant increases. H&E and oil red O staining confirmed hepatic lipid accumulation. These findings collectively underscore that *db*/*db* mice serve as a robust model for MASLD (Figure 1).

### 3.2. Overview of m5C Methylation in the Livers of db/db and NC Mice

**Figure 2:**
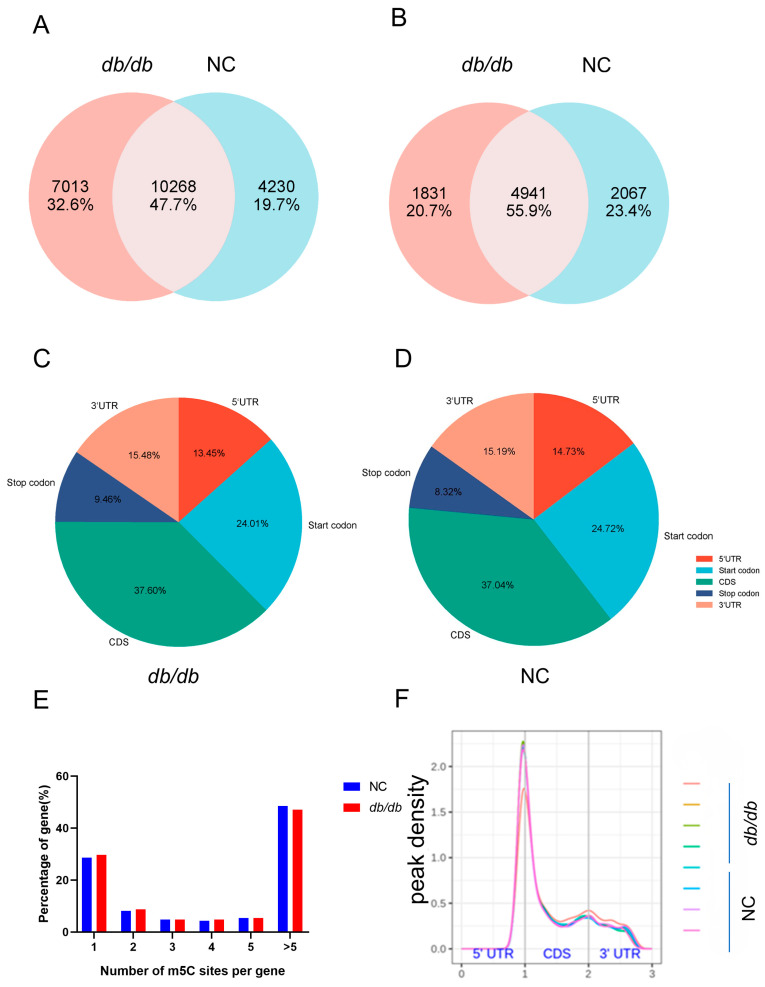
Overview of m5C methylation in the livers of *db*/*db* and NC mice: (**A**) The overlap and differences in m5C peaks between *db*/*db* and NC mice. (**B**) The overlap and differences in m5C-modified genes between *db*/*db* and NC mice. Pie charts showing the percentage of m5C peaks in five nonoverlapping segments of transcripts in (**C**) *db*/*db* and (**D**) NC mice. (**E**) The distribution of genes harboring different numbers of m5C peaks in two groups. (**F**) The pattern of the m5C peak distribution along mRNA lengths in *db*/*db* and NC mice. *n* = 4 biologically independent mice per group.

After RNA was extracted from the liver tissues of *db*/*db* and NC mice, bisulfite sequencing was performed to analyze the 5-methylcytosine modification of RNA. A Venn diagram illustrated that 10,268 m5C peaks were shared between the two groups, while *db*/*db* mice exhibited 7013 unique peaks. Furthermore, 4941 m5C-modified mRNAs overlapped in both groups, with *db*/*db* mice showing 1831 distinct m5C-modified mRNAs (Figure 2A,B). For the precise mapping of m5C sites, peaks were annotated to five mRNA regions: CDS, 5′UTR, 3′UTR, start codon, and stop codon. Analysis revealed that m5C peaks were predominantly concentrated in the CDS (37.04–37.60%) and start codon regions (24.01–24.72%) (Figure 2C,D). Notably, 28.6% of m5C-methylated coding genes in the NC group (and 29.6% in the *db*/*db* group) contained a single m5C peak, and the majority of genes contained more than five peaks (Figure 2E). Subsequent evaluation of m5C peak distribution across the transcriptome revealed a consistent pattern during MASLD progression, with peaks predominantly located in the CDS, stop codon region, and start codon region (Figure 2F).

### 3.3. Distribution and Biological Function of Different m5C Methylations in db/db and NC Mice

#### 3.3.1. The Analysis of Differentially Methylated m5C Sites (DMMSs) in *db*/*db* and NC Mice

Out of the m5C-altered mRNA sequences, we identified 8299 differentially methylated m5C sites. Among these, 4074 methylated sites were specific to the *db*/*db* group (Supplementary Table S2), while 3725 methylated sites were unique to the NC group (Supplementary Table S3). Additionally, 400 sites were methylated in both sample groups, but the methylation levels varied (Supplementary Table S4). The most notable shifts in methylation, both upward and downward, are detailed in Table 1 and Table 2, highlighting the top ten sites with the most significant fold changes observed in MASLD.

**Figure 3:**
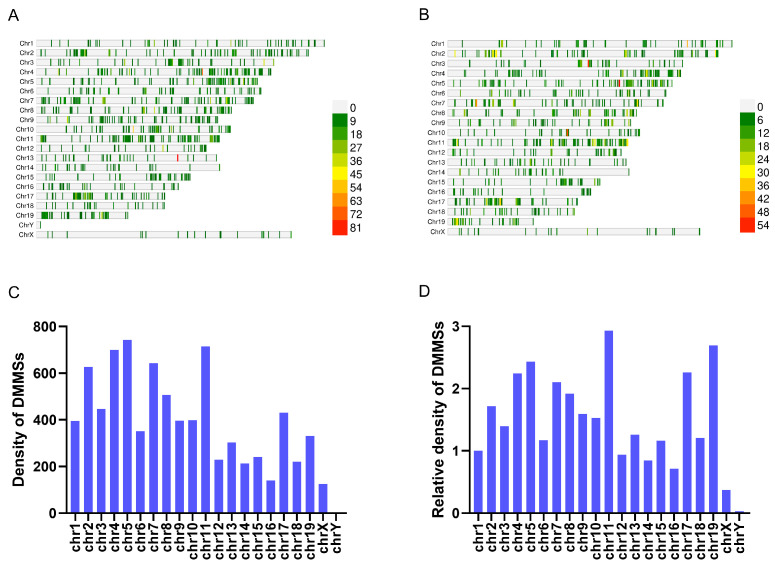
Distribution of differential m5C modification sites: The chromosomal distribution of (**A**) hypermethylated m5C sites and (**B**) hypomethylated m5C sites in mRNA was analyzed and contrasted between *db*/*db* and NC mice. (**C**) The comprehensive mapping of all DMMSs onto specific chromosomes. (**D**) The relative distribution of all DMMSs mapping onto length-normalized chromosomes. *n* = 4 biologically independent mice per group.

All DMMSs were mapped onto specific chromosomes, clearly revealing their distribution patterns (Figure 3A,B). Chromosomes 5, 11, 4, and 7 exhibited the highest absolute number of DMMSs (Figure 3C). Notably, when accounting for chromosome length to assess relative density, chromosomes 19, 11, 7, and 5 emerged as the leading contenders, demonstrating the highest concentration of DMMSs per unit length (Figure 3D).

#### 3.3.2. Biological Function of Differentially Methylated m5C Genes (DMMGs) in *db*/*db* and NC Mice

**Figure 4:**
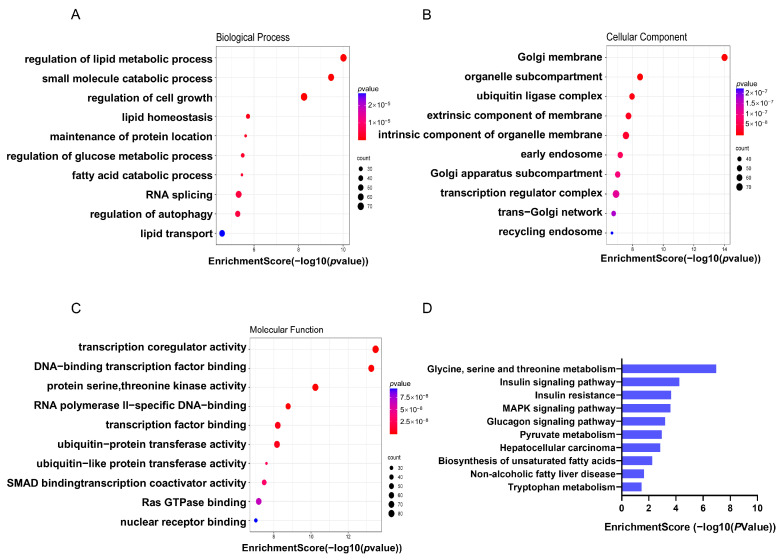
Biological function of DMMGs in *db*/*db* and NC mice: GO analysis was conducted to categorize the DMMGs across three key domains: (**A**) biological processes, (**B**) cellular components, and (**C**) molecular functions. (**D**) KEGG pathway analysis was performed to identify the pathways associated with these DMMGs between *db*/*db* and NC mice. *n* = 4 biologically independent mice per group.

To elucidate the functional implications of genes associated with differentially methylated m5C sites in MASLD, we conducted the Gene Ontology analysis. In terms of biological processes, mRNAs with hypermethylated m5C sites showed a strong connection to lipid metabolism. Upon examining cellular components, these mRNAs were primarily enriched in the nucleus. Regarding their molecular functions, they were significantly associated with ubiquitin protein activity (Figure 4A–C). Moreover, the KEGG pathway analysis emphasized that genes showing m5C hypermethylation in the *db*/*db* group were notably enriched in pathways related to MASLD, suggesting a tight relationship between m5C methylation and MASLD (Figure 4D).

### 3.4. Detection of RNA-Seq-Based Gene Expression Variations in db/db Mice

**Figure 5:**
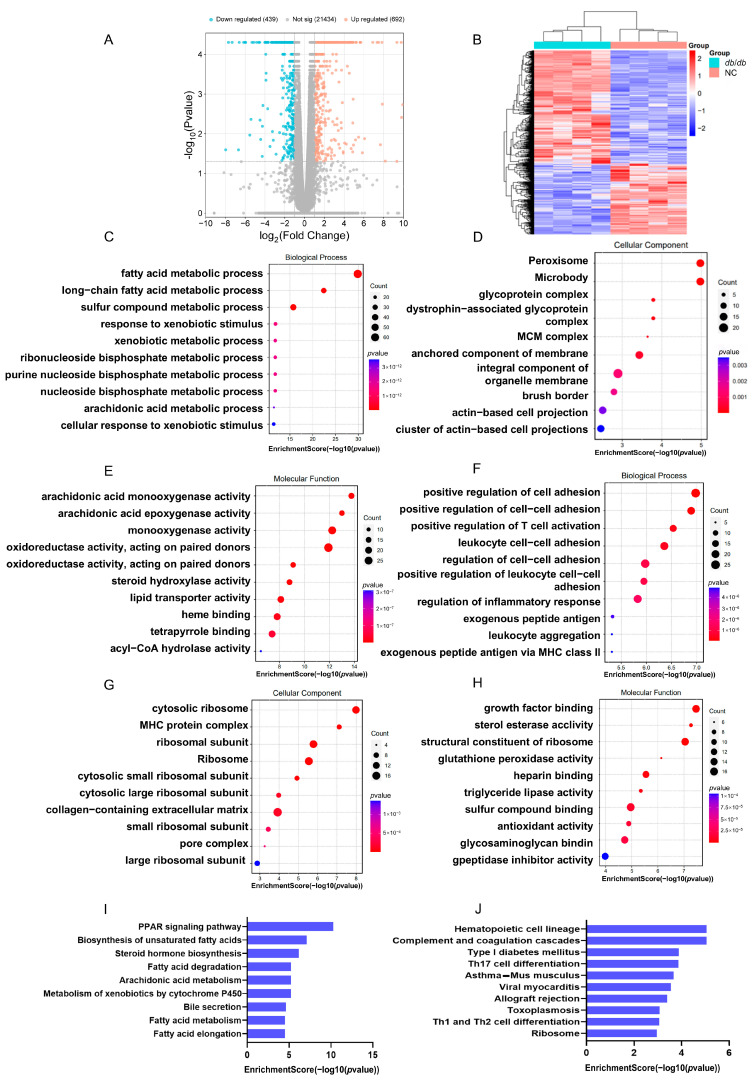
Detection of RNA-Seq-based gene expression variations in *db*/*db* mice: (**A**) Volcano plot illustrating DEGs between *db*/*db* and NC mice. (**B**) Heatmap displaying DEGs in *db*/*db* and NC mice. GO analysis of the upregulated DEGs in *db*/*db* and NC groups in (**C**) biological process, (**D**) cellular component, and (**E**) molecular function categories. GO analysis of the downregulated DEGs in *db*/*db* and NC groups in (**F**) biological process, (**G**) cellular component, and (**H**) molecular function categories. (**I**) Top 10 KEGG pathways with upregulated gene enrichment. (**J**) Top 10 KEGG pathways with downregulated gene enrichment. *n* = 4 biologically independent mice per group.

We used RNA sequencing to identify differentially expressed genes with a fold-change of at least 2 and a *p*-value below 0.05 in the livers of *db*/*db* and NC groups. The analysis uncovered 692 genes that were upregulated and 493 that were downregulated (Supplementary Table S5). Visualized through a volcano plot and supported by cluster analysis, the data highlight a striking divergence in gene expression profiles between the *db*/*db* and NC groups (Figure 5A,B). The top 10 upregulated and downregulated genes are listed in Table 3. To delve into the physiological and pathological implications of DEGs in MASLD, GO analysis and KEGG pathway analysis were carried out. The GO analysis indicated that the upregulated genes were chiefly associated with fatty acid metabolism, peroxisome function, and arachidonic acid monooxygenase activity (Figure 5C–E). Conversely, the downregulated genes were significantly associated with processes such as promoting cell adhesion, enhancing cytosolic ribosome activity, and binding to growth factors (Figure 5F–H). Meanwhile, KEGG pathway analysis revealed upregulated genes predominantly associated with lipid metabolism and downregulated genes connected to hematopoietic cell lineage (Figure 5I,J).

### 3.5. Integrated Analysis of RNA-BS-Seq and RNA-Seq Data

**Figure 6:**
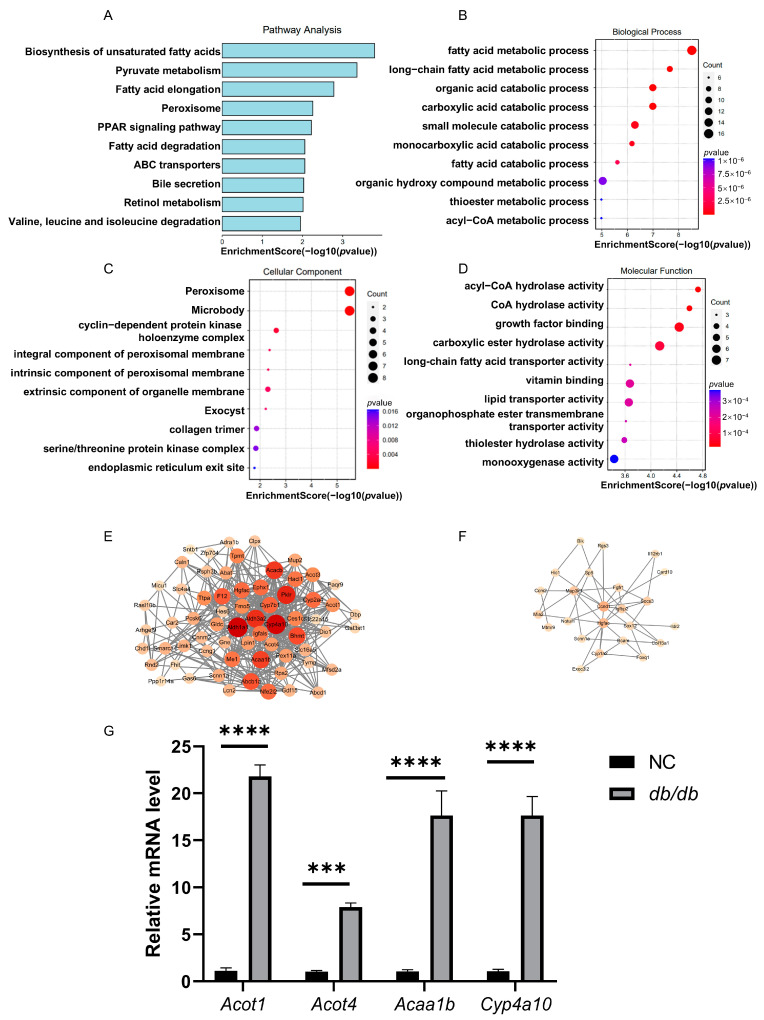
Integrated analysis of RNA-BS-Seq and RNA-Seq data: (**A**) KEGG analysis of mRNA with significant changes in both m5C and mRNA levels between *db*/*db* and NC mice. GO analysis of mRNA with significant changes in both m5C and mRNA levels between *db*/*db* and NC mice in (**B**) biological processes, (**C**) cellular components, and (**D**) molecular functions. (**E**,**F**) Protein–protein interaction network of genes with both altered m5C modification and changed mRNA levels: The left represents m5C hypermethylation sites. The right represents m5C hypomethylation sites. (**G**) Relative mRNA levels of *Acot1*, *Acot4*, *Acaa1b*, and *Cyp4a10* in livers of *db*/*db* and NC mice were determined by RT-qPCR. *n* = 4 biologically independent mice per group. Data are expressed as mean ± SEM. *** *p* < 0.001. **** *p* < 0.0001.

We identified 156 overlapping differentially expressed genes that showed concordant alterations in both mRNA and m5C modification (Supplementary Table S6). Notably, 72 genes exhibited concurrent upregulation, indicating their functional involvement in modulating the mRNA expression of m5C-modified genes. Furthermore, upregulated mRNA and m5C modification levels were observed in more genes than the downregulation of mRNA and m5C modification levels. Among these 156 DEGs, key genes in fatty acid metabolism, such as *Acaa1b*, *Cyp4a10*, *Acot1*, and *Acot4*, were significantly upregulated in both mRNA levels and m5C modification levels. These observations were corroborated by KEGG and GO pathway enrichment analyses, which highlighted substantial enrichment in fatty acid and pyruvate metabolic pathways (Figure 6A–D). Using Cytoscape (version 3.10), we constructed a protein interaction network for genes with both altered m5C methylation and mRNA levels in *db*/*db* mice [34]. This analysis identified *Aldh1a1*, *Cyp4a10*, *Acaa1b*, and *Aldh3a2* as central proteins within the network (Figure 6E,F). The results of RT-qPCR further confirmed that expression levels of *Acot1*, *Acot4*, *Acaa1b*, and *Cyp4a10* were significantly increased in the *db*/*db* group (Figure 6G). Collectively, these findings emphasize the crucial role of these genes and their associated pathways in the protein interaction network and molecular mechanisms underlying MASLD.

## 4. Discussion

This research focused on exploring the impact of m5C RNA methylation on MASLD. Our findings, to our knowledge for the first time, revealed that genes associated with m5C methylation are crucial in hepatic lipid metabolism pathways. Moreover, elevated gene expression correlated with increased m5C methylation levels, indicating a strong association between the two processes.

RNA modifications that occur after transcription are remarkably conserved across evolution, appearing in all domains of life [35]. RNA methylation is the most prevalent post-transcriptional modification of RNA nucleotides, with over 170 documented modifications to date. These modifications critically regulate RNA function and stability [36]. m5C methylation is a widespread modification in eukaryotic mRNA, with transcriptome studies revealing over 10,000 distinct m5C sites [37]. While this modification has also been observed in archaea, it remains notably absent in bacterial mRNA systems [38]. A growing body of research suggests that m5C methylation is not merely a structural feature but functionally significant; it appears to play a pivotal role in various aspects of RNA biology. These include but are not limited to RNA stability, export mechanisms, translational efficiency, and even the intricate process of long-distance RNA transport [25]. Broadly speaking, mRNA modifications primarily function by attracting specific protein partners, a mechanism well documented for m6A-modified RNA [39]. Similarly, m5C modification in mRNA requires specific recognition by binding proteins, including ALYREF and YBX1, to exert its regulatory functions [40,41]. Emerging studies highlight the significance of m5C methylation in adipogenesis. For instance, suppressing NSUN2, a key m5C methyltransferase, promotes lipid accumulation in 3T3-L1 preadipocytes by accelerating cell cycle progression during mitotic clonal expansion [42]. The Aly/REF export factor (ALYREF) facilitates the transport of YBX2/SMO, which exhibits elevated m5C levels, from the nucleus to the cytoplasm. This process enhances the expression of YBX2/SMO protein, ultimately inhibiting adipogenesis while concurrently promoting myogenesis. Genes characterized by hypermethylated m5C were found to be significantly involved in pathways that disrupt adipocyte formation while facilitating muscle development. Laboratory experiments demonstrated that m5C methylation effectively reduced fat storage and stimulated the differentiation of muscle cells [43]. These findings collectively highlight a significant association between m5C modification and lipid metabolism, especially in adipose tissue, emphasizing its crucial biological role in regulating these processes. However, the involvement of m5C modification in hepatic lipid metabolism has yet to be reported in the literature. Current treatment options for MASLD, MASH, and HCC remain limited, underscoring the need for novel therapies. Emerging research on gene expression regulation, particularly through RNA modifications, including m6A, m1A, m5C, and pseudouridine (Ψ), holds promise as a therapeutic strategy. These modifications play critical roles in RNA processing and cellular physiology. By regulating key pathways (e.g., lipid metabolism, fibrogenesis), they offer mechanism-based therapeutic opportunities for hepatic disorders [44]. RNA modifications, through the fine regulation of the expression of genes related to lipid metabolism and inflammation, directly contribute to the pathogenesis of fatty liver. Previous studies have found that changes in m6A and related enzymes are highly correlated with the development of MASLD. m6A methyltransferases METTL3/METTL14 promote the expression of genes related to lipid synthesis (such as *Srebp1* and *Fasn*), exacerbating hepatic steatosis [45]. Overexpression of the demethylase FTO may alleviate fatty liver by reducing the m6A level of lipid metabolism genes [46,47,48]. The reader protein YTHDF2 promotes fat accumulation by degrading mRNAs related to lipid metabolism (such as *Pparα*) [49]. Although m6A is one of the most well-studied RNA modifications, it remains crucial to emphasize its concurrent existence alongside other modifications. Pseudouridine (Ψ) and A-to-I editing drive MASH progression by activating NF-κB signaling [50,51,52]. The elevated expression of m1A methyltransferases (TRMT10C, TRMT6) and ribosomal RNA processing protein 8 (RRP8), along with m5C reader YBX1, demonstrate a significant association with unfavorable prognosis in hepatocellular carcinoma [53,54,55]. Our growing understanding of RNA modification mechanisms is revealing promising opportunities for developing targeted RNA therapeutics in hepatology. This groundbreaking paradigm could transform current treatment modalities, potentially yielding superior clinical outcomes for patients across the spectrum of liver diseases.

The defining feature of MASLD is the excessive accumulation of triglycerides in hepatic cells. This condition, known as steatosis, occurs when hepatic lipid production (via de novo lipogenesis) and fatty acid uptake exceed the liver’s capacity to eliminate them through oxidation or VLDL (very-low-density lipoproteins) transport [56]. This research used a mouse model of MASLD to investigate m5C methylation, uncovering distinct patterns between *db*/*db* and NC groups, which underscored the evolving characteristics of m5C methylation alterations. This study identified differentially methylated mRNAs, revealing their involvement in critical biological processes. Notably, GO and KEGG analyses of genes with differential m5C modification sites demonstrated that up-methylated genes were predominantly linked to lipid metabolism pathways, highlighting the pivotal role of m5C methylation in this process. Because of the central function of the liver in lipid metabolism, these findings reveal a significant connection between m5C methylation and hepatic lipid regulation. This study also reveals distinct m5C modification profiles in *db*/*db* mice compared to NC groups. The integration of RNA-Seq and RNA-BS-Seq data showed a positive correlation between m5C methylation levels and mRNA expression in *db*/*db* mice. Additionally, enrichment analysis revealed a strong association between these changes and fatty acid metabolic pathways, highlighting their importance in hepatic lipid metabolic regulation. Collectively, our results provide compelling evidence that m5C methylation modifications serve as important regulatory factors in MASLD development.

There are several limitations of this study that we would like to point out. First, the sample size of mice was relatively small. Second, we used only one MASLD model, the *db*/*db* mice. As MASLD is a heterogeneous disease, a single model may not be enough to elucidate the underlying mechanism. The relationship between m5C methylation and MASLD needs to be validated and explored in more MASLD models, such as *ob*/*ob*, HFD (high-fat diet)-, or CD-HFD (choline-deficient high-fat diet)-induced mouse models. Third, the molecular mechanism by which m5C methylation influences MASLD remains unclear, and we will explore it in depth in the future.

## 5. Conclusions

m5C methylation plays an important role in post-transcriptional regulation, influencing processes such as alternative splicing and translation. This research highlights a significant link between m5C methylation and fatty liver, suggesting that m5C is a potential therapeutic target. Furthermore, investigating the role of m5C binding proteins in fatty liver may provide a promising avenue for advancing the treatment of MASLD. Our investigation revealed distinct m5C methylation patterns in mouse models of fatty liver compared to controls, underscoring a strong connection between m5C methylation and the regulation of lipid metabolism. These findings establish a foundation for future research aimed at enhancing metabolic function in fatty liver disease.

## Figures and Tables

**Table 1:** The ten leading hypermethylated peaks in *db*/*db* mice.

Chromosome	RefPos	RefStrand	GeneName	Foldchange
19	7516421	+	Atl3	3.577619607
10	76896699	−	Col18a1	3.101572825
19	7516425	+	Atl3	3.090842199
11	118302035	−	Cant1	3.044066762
19	7516426	+	Atl3	2.946495372
10	75773545	+	Chchd10	2.677710514
11	118302029	−	Cant1	2.591881931
2	144112449	−	Snx5	2.581124981
19	38111275	−	Rbp4	2.529759085
10	30076483	−	Cenpw	2.507066041

**Table 2:** The ten leading hypomethylated peaks in *db*/*db* mice.

Chromosome	RefPos	RefStrand	GeneName	Foldchange
4	148543216	+	Mtor	3.739900791
6	94676492	−	Lrig1	3.61000291
19	47568459	+	Slk	3.462946262
12	51737918	+	Ap4s1	3.441410522
3	108191651	+	Sort1	3.301221634
8	123876449	−	Sult5a1	3.264812185
7	127159861	+	Gm42715	3.23104311
1	9818350	−	Vcpip1	3.21986455
14	70526697	−	Ppp3cc	3.197623321
17	31519274	+	Slc37a1	3.164549205

**Table 3:** The top 10 genes show upregulation or downregulation.

Gene Name	Chrome	Strand	Regulation	Fold Change	*p*-Value
Sult3a1	chr10:33863934–33879475	+	Up	980.08	0.00185
Cyp2b13	chr7:26061494–26096196	+	Up	901.21	0.0039
Sult1e1	chr5:87575967–87591611	−	Up	875.72	0.00005
Cyp2b9	chr7:26173410–26210661	+	Up	649.61	0.00005
Sult2a5	chr7:13623966–13670801	+	Up	647.37	0.0499
Hao2	chr3:98874576–98893239	−	Up	287.06	0.0491
Cyp3a41a	chr5:145694059–145720136	−	Up	235.47	0.0018
Sult2a7	chr7:14465158–14492926	−	Up	226.97	0.00005
A4gnt	chr9:99612501–99622367	+	Up	204.94	0.01895
Ly6d	chr15:74762055–74763567	−	Up	179.17	0.00005
Serpina9	chr12:103980242–104013755	−	Down	252.27	0.0255
Rpl21	chr5:146832889–146837032	+	Down	207.83	0.00005
Camk2b	chr11:5969643–6066362	−	Down	159.94	0.00005
Hsd3b5	chr3:98382480–98763128	−	Down	102.79	0.02555
Serpina1e	chr12:103946930–103958975	−	Down	97.10	0.00005
Adh6-ps1	chr3:138374120–138388291	+	Down	70.99	0.00005
Enho	chr4:41569774–41641416	−	Down	64.92	0.00005
BC048546	chr6:128539821–128581606	−	Down	62.24	0.00005
Cib3	chr8:72204334–72212837	−	Down	46.44	0.0108
Gm3839	chr14:11280734–11356726	−	Down	42.66	0.00435

## Data Availability

The original contributions presented in this study are included in the article. Further inquiries can be directed to the corresponding authors.

## Supplementary Materials

The following supporting information can be downloaded at https://www.mdpi.com/article/10.3390/cimb47050305/s1. The datasets generated in this study can be found in online repositories.

## Abbreviations

The following abbreviations are used in this manuscript:

DMMGs	Differentially methylated m5C genes
DMMSs	Differentially methylated m5C sites
HCC	Hepatocellular carcinoma
MASH	Metabolic dysfunction-associated steatohepatitis
MASL	Metabolic dysfunction-associated steatotic liver
RNA-BS-Seq	RNA bisulfite sequencing
RNA-Seq	RNA transcriptome sequencing
m5C	5-methylcytosine
MASLD	Metabolic dysfunction-associated steatotic liver disease
H&E	Hematoxylin and eosin
KEGG	Kyoto Encyclopedia of Genes and Genomes
GO	Gene Ontology
DEGs	Differentially expressed genes
