# Supplementary material for: Comprehensive Analysis of Differentially Expressed Profiles of mRNA 5-Methylcytosine Modification in Metabolic Dysfunction-Associated Steatotic Liver Disease
Source: Curr Issues Mol Biol. 2025 Apr 26;47(5):305. doi: 10.3390/cimb47050305 (PMC12110074; doi:10.3390/cimb47050305)
Supplement: Supplementary file 1 [file cimb-47-00305-s001.zip › Supplementary Table S6.pdf]

Supplementary Table S6. Genes that exhibit a significant change in both m5C level and mRNA transcript abundance in *db/db* mice compared with normal mice

Types of DEGs	Gene Names
Hyper-up (n=72)	Rnd2;Aldh3a2;Acaa1b;Clpx;Nfe2l2;Micu1;Ccng1;Rasl10b;Acot3;Tpmt;Hacl1;Brd1;Tymp;Abca3;Gldc;Mmp19;Arhgef9;Lcn2;Car2;Fmo5;Chd1l;Col15a1;Mfsd2a;Ccng2;Limk1;Exoc4;Pcsk6;Pex11a;Oat;Abcd1;Gas6;Tenm3;Smarca4;Col12a1;Me1;Topbp1;Slc22a15;Ccdc122;Nubpl;Gdf15;Ephx1;Ppl;Zfp704;Trim7;Abcb1a;Ildr2;Pklr;Acacb;Zfp532;Acot6;Slc16a5;Ermp1;Dcun1d3;Gal3st1;Fam171a1;Adra1b;Acot4;Aldh1a1;Abat;Caln1;Sntb1;Fhit;Slc4a4;Cnnm2;Paqr9;Cyp4a10;Acot1;Ttpa;Cyp2a4;Bhmt;Cenpw;Fam229a
Hyper-down (n=25)	Col5a3; Lpin1; F12; Rsph3b; Ttc39c; Il1r1; Gne; Hgfac; Chic2; Arpc1b; Scnn1a; Card10; Dio1; Ppp1r14a; Josd2; Cyp7b1; Ubald1; Rps2; Igfals; Ces1c; Dbp; H2-Q7; Hes6; Nrbp2; Mup2
Hypo-up (n=19)	Bcam; Map3k1; Synj2; Gldc; Dennd2d; Col15a1;4931406C07Rik; Vat1; Mtmr9; Mia2; Ildr2; S1pr5; Basp1; Ppp1r3b; Zmat1; Atad2b; Slc27a4;1110002L01Rik; Mup19
Hypo-down (n=40)	Ccm2; Il12rb1; Cyp51; Dnase2a; Exoc3l2; Bik; Ppm1m; Ttc39c; Mcm10; Enho; Gba2; GneSlc25a33; Hgfac; Scnn1a; Fgfr1; Cyp1a2; Card10; Kri1; Foxq1; Susd4; Josd2; Rpl12; Igfbp2; Ubald1; Fam222a; Hps4; Notum; Hic1; Sox12; Socs3; Rgs3; Apol9b; Sort1; Selenbp2; Ccnd1; Sp5; Nrbp2; Adh6-ps1; C2cd4d
