# Supplementary material for: Comprehensive Analysis of Differentially Expressed Profiles of mRNA 5-Methylcytosine Modification in Metabolic Dysfunction-Associated Steatotic Liver Disease
Source: Curr Issues Mol Biol. 2025 Apr 26;47(5):305. doi: 10.3390/cimb47050305 (PMC12110074; doi:10.3390/cimb47050305)
Supplement: Supplementary file 1 [file cimb-47-00305-s001.zip › Tables legends.pdf]

Table S1: The primer sequences for RT-qPCR,

Table S2: The *db/db* group-specific methylated sites,

Table S3: The NC group-specific methylated sites,

Table S4: The sites of the *db/db* group and NC group with different methylation levels,

Table S5: The upregulated and downregulated genes in the *db/db* mouse liver,

Table S6: DEGs changing in both mRNA and m5C modification.
